# Joule Heating in Controlled Atmospheres to Process Nanocarbon/Transition Metal Oxide Composites and Electrodes

**DOI:** 10.1021/acsanm.4c02081

**Published:** 2024-06-14

**Authors:** Shegufta Upama, Luis Arevalo, Afshin Pendashteh, Anastasiia Mikhalchan, Micah J. Green, Juan Jose Vilatela

**Affiliations:** † Department of Materials Science & Engineering, Texas A&M University, College Station, Texas 77843, United States; ‡ IMDEA Materials Institute, Getafe, Madrid 28906, Spain; § Artie McFerrin Department of Chemical Engineering, Texas A&M University, College Station, Texas 77843, United States

**Keywords:** carbon nanotube fabric, inorganic, metal oxide, Joule heating, direct current, nanostructured network, composite

## Abstract

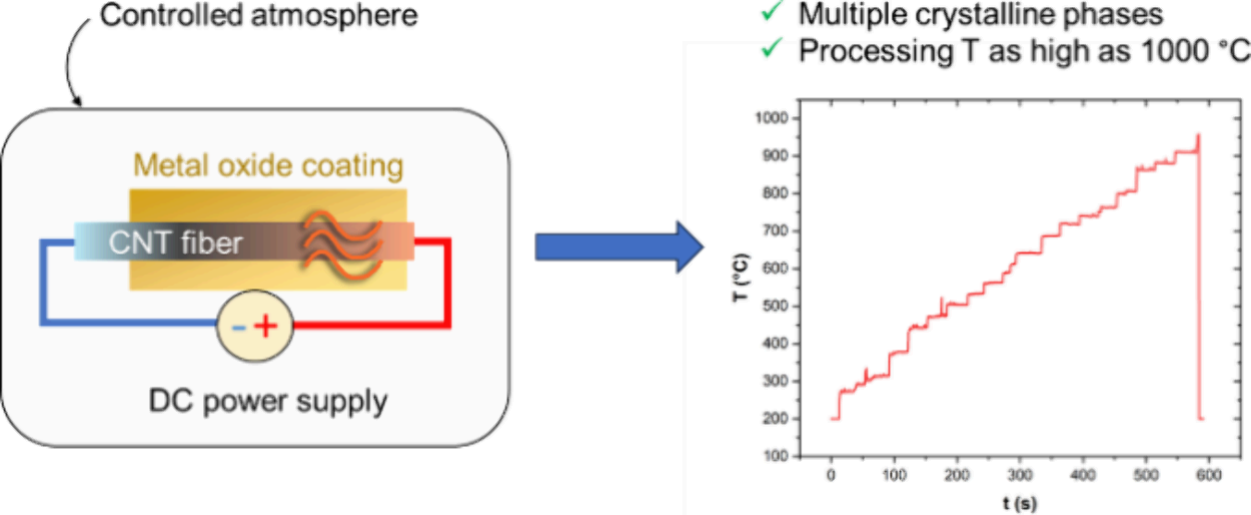

Composites of nanocarbons and transition metal oxides combine excellent mechanical properties and high electrical conductivity with high capacitive active sites. These composites are promising for applications such as electrochemical energy conversion and storage, catalysis, and sensing. Here, we show that Joule heating can be used as a rapid out-of-oven thermal processing technique to crystallize the inorganic metal oxide matrix within a carbon nanotube fabric (CNTf) composite. We choose manganese oxide and vanadium oxide as model metal oxides and show that the Joule heating process is rapid and enables accurate control over the temperature and phase transitions. Next, we use thermogravimetric analysis and Joule heating experiments in controlled atmospheres to show that metal oxides can actually catalyze thermal degradation and reduce the thermal stability of the CNTs, which could limit processing of many oxides. We solve this by using a reducing hydrogen atmosphere to successfully extend the Joule processing window and thermal stability of the CNTf/metal oxide composite to ∼1000 °C.

## 1 Introduction

Transition metal oxides and their nanocarbon composites have applications in a wide range of fields, such as electrochemical energy conversion and storage (fuel cells, solar cells, supercapacitors, lithium ion batteries),^1−4^ catalysis,^5−8^ and sensing.^9,10^ Nanocomposites of these inorganics and carbon nanotubes (CNTs) are particularly important due to their excellent electrochemical performance, cycling stability, and low toxicity.^11−13^ However, in order to maximize their performance as electrodes, the CNTs should be connected to each other to form a conductive network, and the inorganic metal oxide should be uniformly distributed within the composite.^14^ Nonwoven, unidirectional CNT fabrics (CNTfs) are a good example of such conductive networks, providing a tough scaffold for the metal oxides and acting as a built-in current collector.

In our prior work, we showed that CNTf can be used both as a metal-free current collector and as a scaffold for growing inorganic phases like molybdenum sulfide (MoS_2_) to form a nanostructured composite with application as a flexible battery electrode.^15^ The electrochemically active inorganic materials coats uniformly onto the CNT network, which provides mechanical reinforcement and a low resistance to charge transfer across the CNT-inorganic interface. Additionally, we utilized the underlying electrically conductive CNT network to generate current-induced heating within the CNTf and, in turn, the composite, as a rapid out-of-oven thermal processing technique to crystallize MoS_2_.^16^

Now, we seek to demonstrate that this Joule heating approach is a general processing route, which can be extended to multiple inorganic matrix materials including those that require higher processing temperatures, like metal oxides, using vanadium oxide (VO_*x*_) and manganese oxide (MnO_*x*_) as model metal oxides.

Since our aim is to push the Joule heating processing limits for CNTf/inorganic composites, it is helpful to first understand the processing limits for the CNT building blocks. The maximum current density and Joule breakdown of individual CNTs,^17^ acid-doped CNT fibers,^18^ and CVD-grown CNT fibers^19^ have been explored before, and the current density at failure was found to be correlated with the maximum temperature of the CNTs before breakdown. In an inert atmosphere such as argon or nitrogen, the CNTs can be Joule-heated to higher temperatures (>1000 °C) than in air, where they oxidize at ∼600 °C. This means that at sufficiently high temperatures, the CNTf will fail, even in inert conditions, so there is an upper limit of current density and temperature that is attainable for this approach. The presence of metal oxides may actually alter this upper limit of Joule heating; Aksel and Eder have demonstrated using conventional oven heating that the presence of metal oxides can catalyze the oxidation of CNTs, and that this effect is dependent on the atmosphere used.^20^ Note that none of these prior studies have utilized Joule heating for inorganic/CNT composite processing or determined how the inorganic phase interacts with the CNT during Joule heating.

Therefore, in this work, we show not only that Joule heating can be applied to out-of-oven manufacturing of inorganic/CNTf composites but also evaluate how the presence of the metal oxide affects CNT thermal stability. By carrying out thermogravimetric analysis and parallel experiments (both in oven and via Joule heating) in controlled atmospheres, we find that the CNTf oxidation onset temperature is decreased by the presence of the inorganic matrix, not only in air but even in inert conditions. This effect can be counteracted by introducing a reducing H_2_ atmosphere, which allows for an extended thermal processing window. This report is the first to uncover this coupling between the inorganic matrix and the CNT network stability for thermal composite processing.

## 2 Experimental Section

### 2.1 Materials

Toluene, ferrocene, thiophene, vanadium(IV) oxide sulfate hydrate (99.99%), manganese(II) nitrate hydrate (99.99%), sodium nitrate (99%), and aluminum (99%). All chemicals were purchased from Sigma-Aldrich and used without further purification.

### 2.2 Methods

#### 2.2.1 Synthesis of Directly Spun CNT Fabrics (CNTfs)

The CNT fabrics were synthesized directly from the gas phase using chemical vapor deposition (CVD). The precursors for CNT growth were toluene (C source), thiophene (promoter), and ferrocene (iron catalyst). These were introduced from the top of a vertical furnace at 1300 °C and a hydrogen atmosphere. As mentioned in our prior work, these synthesis conditions were chosen to produce bundles of few-layer CNTs.^21^ The CNTs were collected as bundles from the bottom of the furnace, taking advantage of the van der Waals attractions between them. These CNT bundles were collected onto a rotating drum for 20 min to form a nonwoven, unidirectional fabric (CNTf).

#### 2.2.2 Synthesis of the CNTf/VO_*x*_ Composite

The pristine CNTf was functionalized using a gas-phase ozone treatment (Jelight UVO-cleaner model 18, U.S.) for 20 min (10 min on each side). This treatment turns the fabric hydrophilic and ensures wetting in the next step,^22^ where VO_*x*_ was deposited onto the treated fabric using electrochemical deposition in a three-electrode setup. The working, reference, and counter electrodes were the treated CNTf, a saturated calomel electrode, and a platinum mesh, respectively. The electrolyte solution consisted of 0.1 M vanadium(IV) oxide sulfate in deionized water, which was maintained at 60 °C under constant stirring. The deposition technique used was chronoamperometry, where a constant potential of 1.8 V was applied for 10 s followed by rest for 20 s, and this cycle was repeated until the total deposition time was 15 min. Afterward, the samples were thoroughly washed in water and ethanol and then dried under ambient conditions.

#### 2.2.3 Synthesis of the CNTf/MnO_*x*_ Composite

The pristine CNTf was ozone-treated as mentioned above. Next, MnO_*x*_ was deposited onto the treated fabric using electrochemical deposition in a three-electrode setup. The working, reference, and counter electrodes were the treated CNTf, a Ag/AgCl (3 M) electrode, and a platinum mesh, respectively. The electrolyte solution consisted of 0.05 M manganese(II) nitrate and 0.1 M sodium nitrate in deionized water. A constant current of 300 μA cm^–2^ was applied for 1 h. As before, the samples were thoroughly washed in water and ethanol and then dried under ambient conditions.

#### 2.2.4 Processing of the CNTf/Metal Oxide Composite

The direct current (DC) heating setup was inside a vacuum chamber (Pfeiffer Vacuum) that was maintained under an air or a vacuum/argon atmosphere (∼3.10 mbar). The former was maintained by keeping the setup open to the atmosphere. The latter was maintained by first evacuating the chamber and then continuously feeding argon to provide an inert atmosphere. The samples (either CNTf/metal oxide or CNTf) were cut using sharp scissors into rectangular strips (0.3 cm × 2.0 cm) and connected to alligator clips along the middle of either end of the fabric. Then, the samples were connected in series to a DC power supply (Delta Elektronika SM660-AR-11) and a 3 Ω resistor, which was added as a safety precaution to limit the current flowing through the sample and prevent sample damage. Two digital multimeters (Keysight 34465A) were used to measure the voltages of the resistor (*V*_*resistor*_) and the DC power supply (*V*_*source*_). A LabView program was used to plot and analyze the voltage and current of the sample according to the following equations:
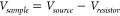
1
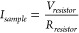
2

The DC voltage was manually modulated at a rate of ∼50 °C/min until the sample reached the target temperature (130, 350, or 550 °C for MnO_*x*_ and 300, 350, or 400 °C for VO_*x*_), and the temperature was maintained for 10 min. Ramping up the DC voltage led to an instant rise in temperature, which was monitored using an infrared pyrometer (Optris CTlaser pyrometer).

For the DC heating setup in hydrogen, we used high-vacuum fittings (Swagelok) to connect a custom-built quartz tube to argon and hydrogen gas lines (see Figure S1). The quartz tube had an additional opening that was connected to a DC power supply via two tungsten rods. A pyrometer (having measurement limits of 200–1500 °C) was used to monitor the temperature of the sample. The setup was first purged with argon, and then the hydrogen valve was turned on, making sure that the hydrogen concentration was below the flammability limit in inert. DC heating was done by modulating the DC voltage under 5% hydrogen and 95% argon flow (total gas flow was 100 SCCM).

#### 2.2.5 Characterization

The morphology and crystalline phase of the samples were characterized using field-emission scanning electron microscopy (FESEM, FEI Helios NanoLab 600i), Raman spectroscopy (Renishaw, fitted with a 532 nm laser source), powder X-ray diffraction (PXRD, Cu Kα radiation, Empyrean, PANalytical Instruments), and wide-angle X-ray spectroscopy (WAXS, Anton Paar). The fracture propagation of the material was followed *in situ* during Joule heating using an optical microscope (Bresser). The sample was connected to a DC power supply using alligator clips and then mounted onto the microscope stage for viewing.

The composite mass fraction and mass loss with temperature was measured using thermogravimetric analysis (TGA Q50, TA Instruments) in air, using a sequential temperature program. First, the temperature was raised from room temperature to 100 °C with 10 °C/min and a dwell time of 20 min, making sure to remove physically adsorbed moisture. Then, the temperature was ramped at 10 °C/min up to 1000 °C with no dwell time. The measurements were taken under two different atmospheres, namely, air and nitrogen. A third atmosphere, namely, hydrogen, was tested using a tube furnace with continuously flowing hydrogen gas. For this last case, only the initial and final masses were measured using a high-precision mass balance. The initial mass was normalized with respect to the average water loss at 100 °C.

The nominal current density was calculated by dividing the current generated in the sample by the cross-sectional area. The lateral dimensions of the fabric was measured using a ruler, while the thickness was measured using a high-precision micrometer. The relative resistance change was calculated by divided the resistance by the initial resistance, *R*_0_, which was defined as the resistance measured at room temperature at time 0.

## 3 Results

**Figure 1:**
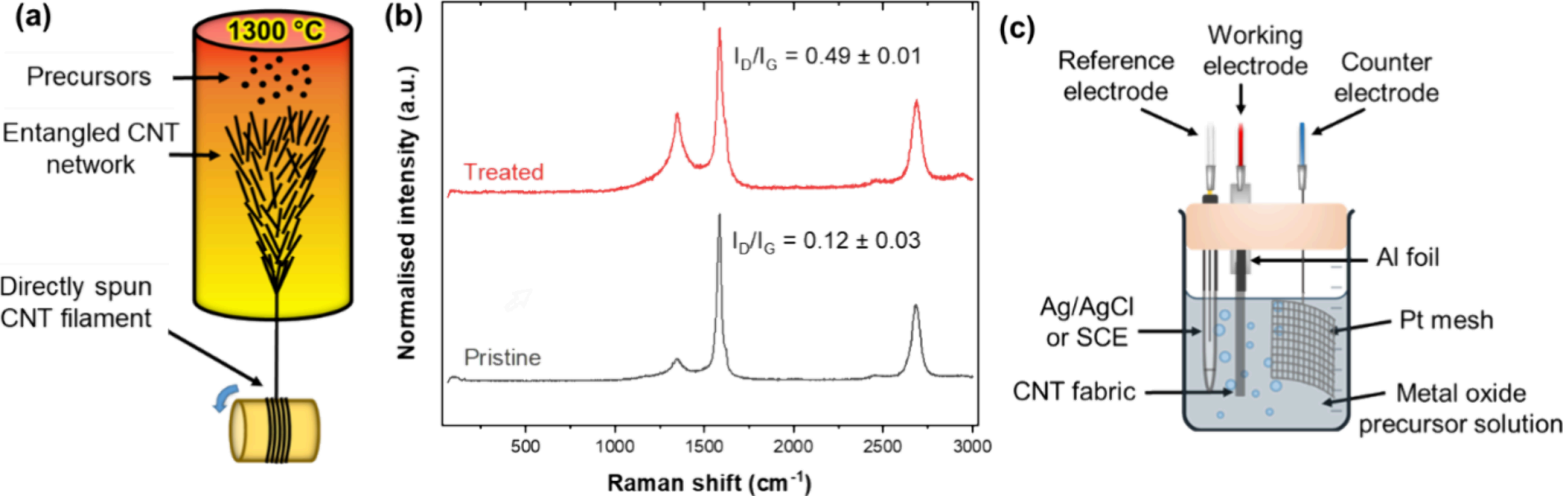
Schematic of the techniques used to fabricate CNTf/metal oxide composites. (a) Synthesis of CNT and CNTf directly from the gas phase using floating catalyst chemical vapor deposition (FC–CVD). (b) Raman spectra of the CNTf before (black spectrum) and after (red spectrum) ozone treatment. (c) Electrochemical deposition of the metal oxide using a three-electrode setup.

The syntheses of the CNTf/metal oxide composites are carried out as follows: We utilize our previously established floating catalyst method (Figure 1a) to produce CNT fabrics of around 100 cm^2^. These CNTf samples are then ozone-treated to create oxygen-containing functional groups (such as, C–O, C=O, and O–C=O)^22^ and improve wettability (Figure 1b); the Raman intensity ratio of the D/G bands increases from 0.12 ± 0.03 to 0.49 ± 0.01, indicating that the ozone functionalization is successful. Finally, the target metal oxide (MnO_*x*_ or VO_*x*_) is deposited onto the functionalized CNTf using electrochemical deposition in an aqueous solution. These samples are the starting point for the Joule heating experiments below.

**Figure 2:**
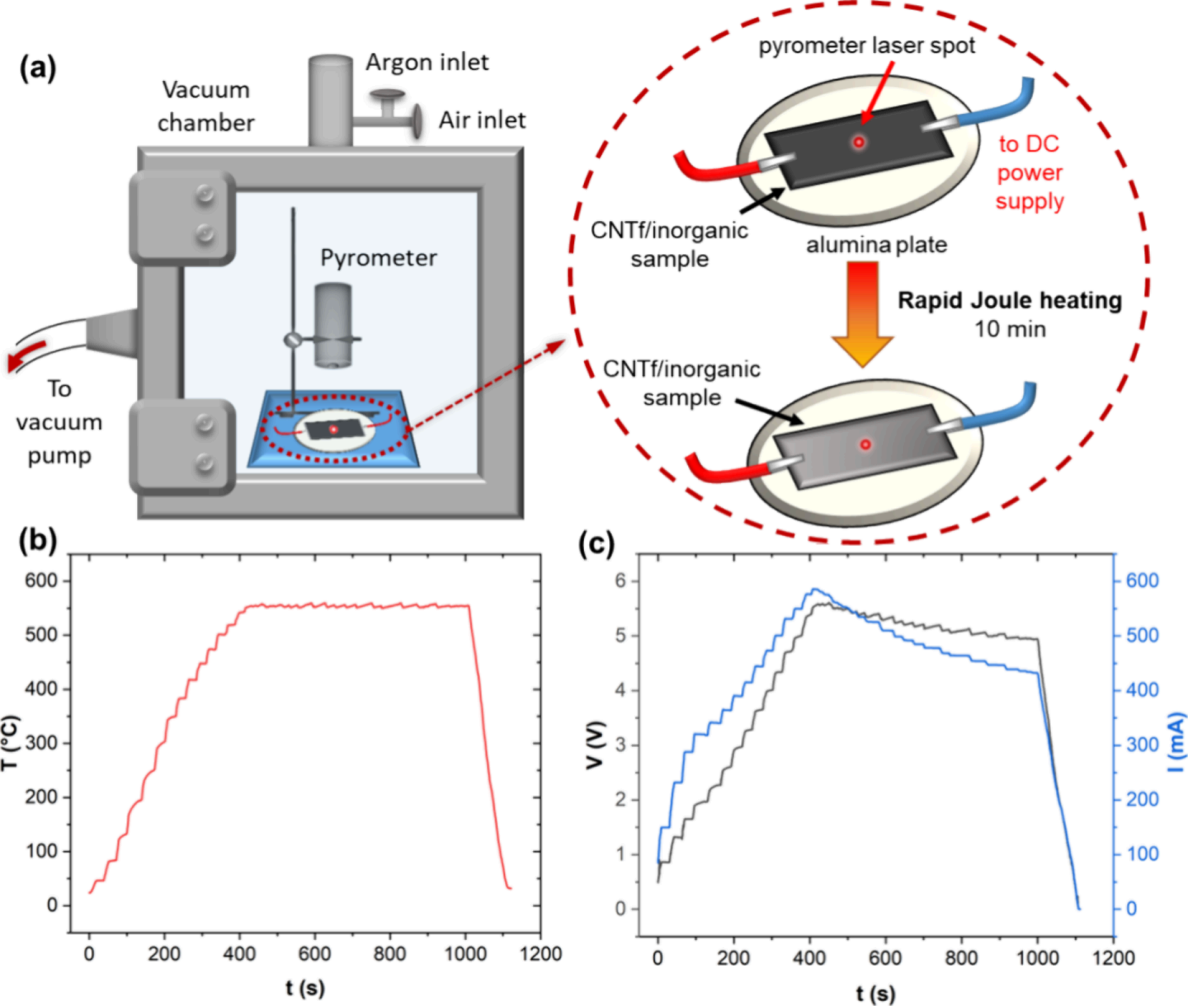
(a) Schematic of the DC heating setup, with a zoomed-in view of the sample showing electrical connections to the DC power supply. After DC heating, the gray-black sample turns silvery, indicating a phase transition of the CNTf/MnO_*x*_ from ε-MnO_2_ to α-Mn_3_O_4_. (b) Temperature profile of a typical DC heating experiment in argon. (c) Voltage and current vs time. The DC voltage was ramped in stages until the target temperature (550 °C) was reached. The voltage was then modulated to maintain the temperature at 550 °C for 10 min.

To carry out Joule heating in a controlled atmosphere, we utilize the setup shown in Figure 2a. We monitor temperature and current as a function of time in response to an input voltage ramp. The voltage is modulated to hit the target temperature which ranges from 130 to 550 °C for MnO_*x*_ and from 300 to 400 °C for VO_*x*_. These temperatures were selected to induce the desired crystalline phase transitions from the as-deposited amorphous phase.^23^ A typical Joule heating experiment is shown in Figure 2b,c for a CNTf/MnO_*x*_ sample in argon. In this case, the temperature and current stabilize rapidly after every input voltage change, both at the heating ramp and as the sample is held at the target temperature (550 °C in this case) for 10 min to crystallize the as-deposited ε-MnO_2_ to α-Mn_3_O_4_. The changes in resistance provide more qualitative insights into the structural changes occurring during processing. The increase in resistance upon heating has a component from the metallic behavior of the CNTs onto which a further increase occurs due to the transformation of the oxide, leading to a parabolic resistance increase during heating and a slow resistance increase upon isothermal treatment (Figure S2). During cooling, resistance decreases with a lower slope and reaches a higher value at room temperature, which is attributed to residual strain on the piezoresistive CNT fiber induced upon cooling.

Joule heating provides a rapid, out-of-oven thermal processing approach. This is particularly important for roll-to-roll processing, where large cumbersome conventional ovens are not desired. In contrast, Joule heating has already been shown to allow for *in situ* heating through simple electrical contacts. A similar technique has been used to partially cure carbon fiber/epoxy structures using Joule heating to make prepregs.^24^ In addition, the throughput time is much faster (time up to temperature) is much faster in Joule heating (<10 min) vs a conventional heating (hours). Temperature ramp occurs in less than 5 s, in contrast to our oven’s typical ramp time of ∼10 min.

**Figure 3:**
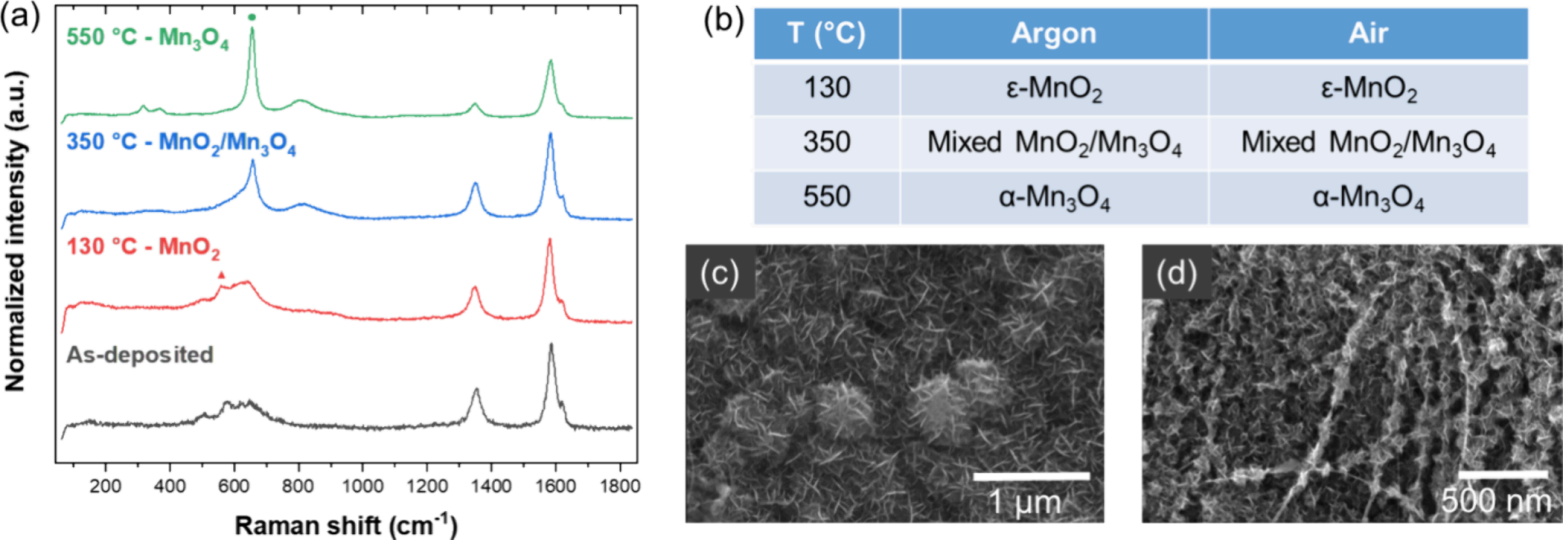
(a) Raman spectra for CNTf/MnO_*x*_ before and after Joule heating to different temperatures. The red triangle corresponds to the ε-MnO_2_ phase, while the green asterisk corresponds to the α-Mn_3_O_4_ phase. (b) Summary of phase transitions of MnO_*x*_ in air and argon environments. (c) SEM image of as-deposited CNTf/MnO_*x*_ with nanoflower morphology. (d) SEM image of CNTf/MnO_*x*_ after indirect DC heating at 550 °C, showing smaller nanoflowers.

Using this approach, we then explore how temperature and atmosphere affect the phase transition of our CNTf/inorganic composites. For the case of MnO_*x*_, the as-deposited phase is amorphous ε-MnO_2_, but the desired crystalline phase is α-Mn_3_O_4_. To induce the phase transition, we varied temperature (130, 350, and 550 °C) and atmosphere (air vs argon), all for 10 min of exposure time. Note that the lower two temperatures were achieved by Joule heating the samples directly, whereas the high temperature was achieved by Joule heating the sample indirectly using a CNT fabric support, which will be explained below. The Raman spectra (Figure 3a) show that the as-deposited amorphous ε-MnO_2_ crystallizes at 130 °C. Heating to 350 °C gives a mixture of two crystalline phases, which result in a broad Raman peak that can be deconvoluted to the ε-MnO_2_ peak at ∼560 cm^–1^ and the α-Mn_3_O_4_ peak at ∼660 cm^–1^. Further heating to 550 °C crystallizes all the MnO_*x*_ to the α-Mn_3_O_4_ phase. The same phases are observed in both air and argon atmospheres; these results are summarized in Figure 3b. The outcomes at the lower temperatures match the calcination study done by Augustin et al., which investigated MnO_*x*_ phase behavior in air and argon using conventional heating.^22^ However, at 550 °C, they reported the α-Mn_2_O_3_ phase, while our Raman peaks (314, 366, and 656 cm^–1^) can be indexed to the α-Mn_3_O_4_ phase.^23,25^ The MnO_*x*_ coats the outside of the network of CNT bundles with nanoflower morphology. The as-deposited phase (Figure 3c) resembles nanoflowers of diameter 311 ± 11 nm, which get dehydrated and calcined at higher temperatures to show small, crystalline nanoflowers of diameter 89.6 ± 12.2 nm on the CNT surface (Figure 3d). Note that the atmosphere did not make a major difference in the resulting structure.

**Figure 4:**
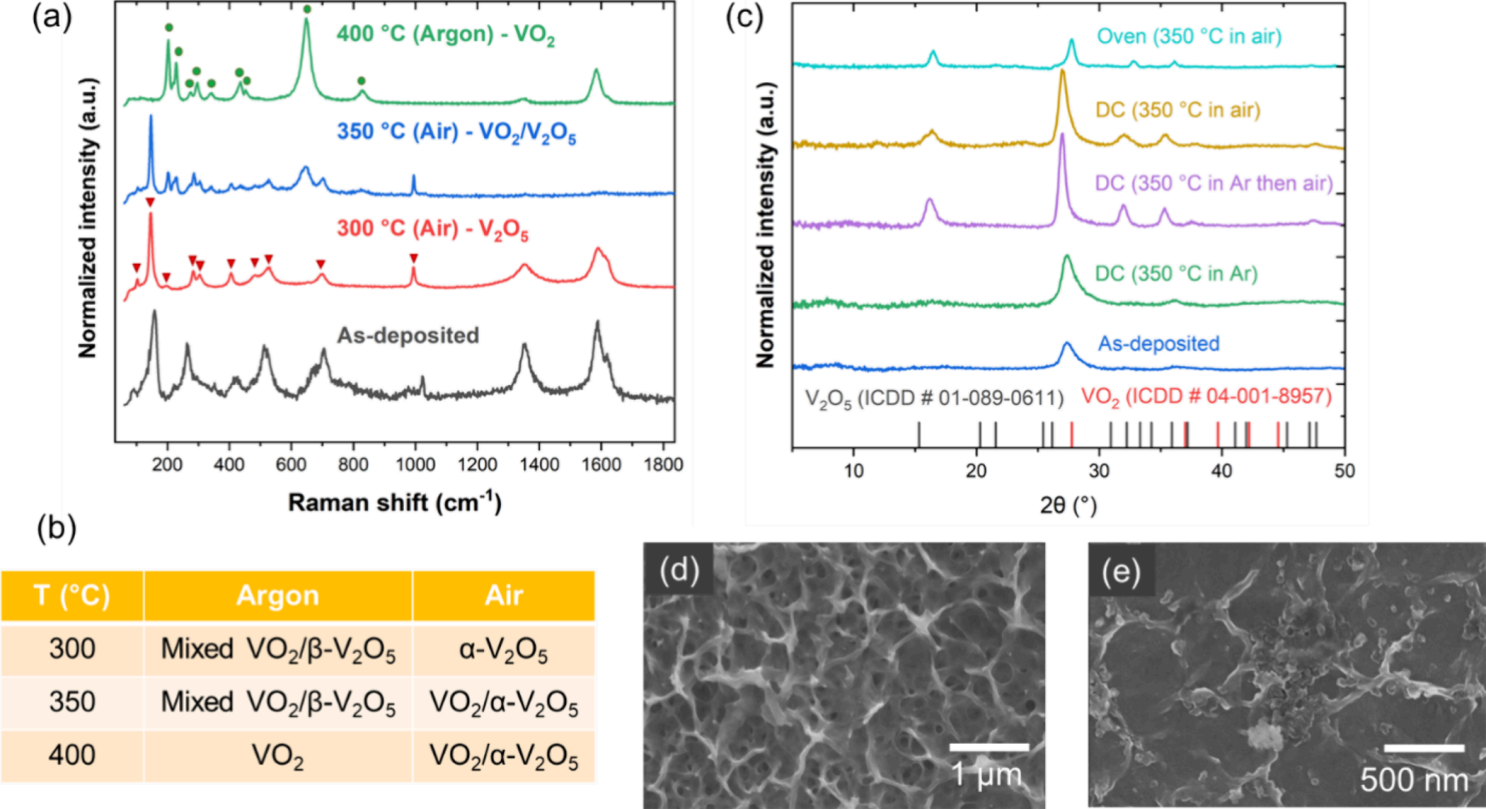
(a) Raman spectra for CNTf/VO_*x*_ before and after Joule heating to different temperatures. The red triangles correspond to the α-V_2_O_3_ phase, while the green circles correspond to the VO_2_ phase. (b) Summary of phase transitions of VO_*x*_ in air and argon environments. (c) Comparison of WAXS spectra for CNTf/VO_*x*_ samples that were DC-heated in air, argon, or a combination of the two environments, along with reference peaks. (d) SEM image of as-deposited CNTf/VO_*x*_, with a porous conformal coating morphology. (e) SEM image of CNTf/VO_*x*_ after DC heating at 350 °C, showing compact, sintered coating.

We carried out similar studies on VO_*x*_, where the as-deposited phase is VO_2_/β-V_2_O_5_ but the desired crystalline phase is α-V_2_O_5_. Again, the temperature and atmosphere were varied. In contrast to MnO_*x*_ (where temperature was the key factor), here the atmosphere played a much stronger role. The Raman spectra (Figure 4a, summarized in Figure 4b) show that the as-deposited VO_2_/β-V_2_O_5_ crystallizes to the α-V_2_O_5_ phase when heated to 300 °C in air. Further heating to 400 °C in air gives a mixed VO_2_/α -V_2_O_5_ phase. In contrast, there is no phase transition from the as-deposited VO_2_/β-V_2_O_5_ phase when heated up to 350 °C in argon. Heating to a higher temperature (400 °C) in argon gives the VO_2_ phase. To distinguish the phase transitions or lack thereof in these two atmospheres, we utilized WAXS to confirm the effect of the atmosphere, particularly around 350 °C. (raw WAXS data shown in Figure S3). According to Figure 4c, when the as-deposited VO_2_/β-V_2_O_5_ is DC heated in argon to 350 °C, there is no phase change because we can see the same peak at 27.28°, which can be indexed to the (011) plane for tetragonal VO_2_. However, if the atmosphere is switched from Ar to air halfway at the 5 min mark during Joule heating, then the orthorhombic α-V_2_O_5_ phase does appear. This shows that the presence of oxygen in the system results in the orthorhombic phase. Accordingly, the same phase transition is observed when the sample is heated in air using either Joule heating or oven heating, verifying that the atmosphere plays a key role in the phase transitions. Note that the peaks for oven heating are red-shifted by ∼0.8° relative to the peaks for DC heating, which corresponds to a relatively larger interplanar distance for the DC-heated sample. As for the morphology of VO_*x*_, the as-deposited phase forms a porous conformal coating around the CNT bundles (Figure 4d). After DC heating (to 350 °C), we get a compact coating of sintered nanocrystalline particles (Figure 4e).

We next aim to assess whether the presence of metal oxides affects the thermal stability of CNTs during Joule heating. This stems from the interest in expanding the Joule heating processing window to high temperatures, and the observation of unexpected thermal degradation of CNT fabric/metal oxide composites under some processing conditions. For example, we compared a pristine CNTf sample and a CNTf/MnO_*x*_ sample that were Joule heated to failure in argon. The CNTf sample reaches 835 °C before failure, while the CNTf/MnO_*x*_ sample fails at a much lower temperature of 542 °C. (Thermal data and SEM images shown in Figures S4 and S5.) The SEM images show evidence of CNT fiber breakage and clusters of carbonaceous structures produced at high temperatures and indicative of local temperature in excess of 1000 °C. Note that the maximum temperature reached by CNTf/MnO_*x*_ before material failure is less than the target temperature for crystallizing α-Mn_3_O_4_ (550 °C). Accordingly, we used an indirect heating setup (where the CNTf/MnO_*x*_ sample was placed on top of a CNTf substrate, and the latter was connected to the DC power supply) to achieve higher temperatures for CNTf/MnO_*x*_ in Figure 3 above.

In an air or Ar atmosphere, the failure in composites during Joule heating occurs rapidly through propagation of a glowing crack across the sample. Close inspection of the CNTf materials and the composites did not show compositional or structural inhomogeneities that could explain this effect or the location of this propagating crack. However, the type of metal oxide clearly affected the onset of this effect.

**Figure 5:**
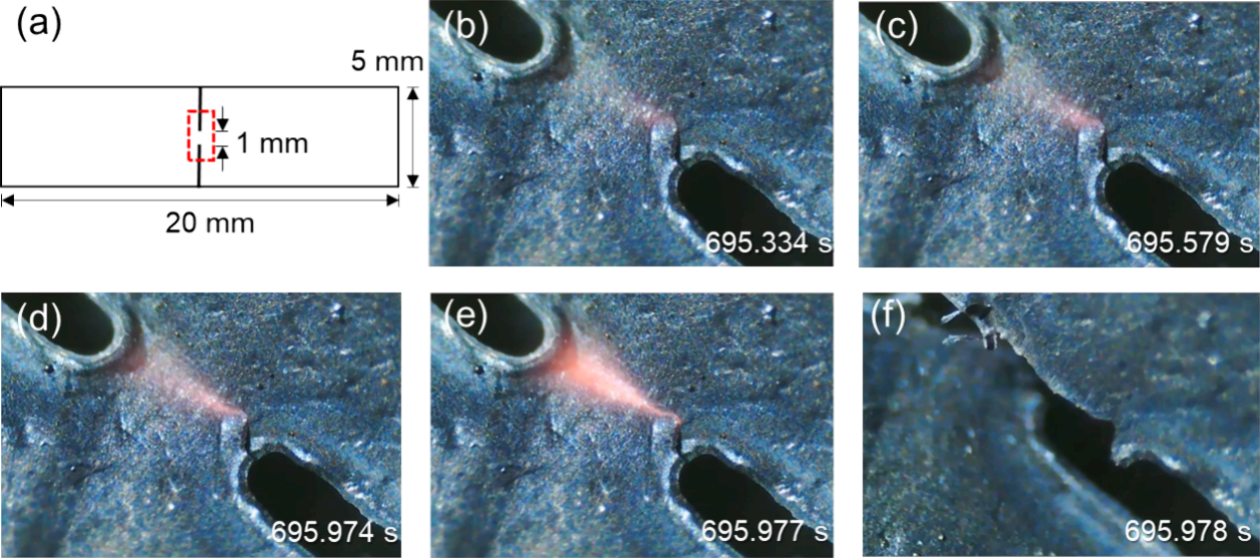
(a) Dimensions of the notched CNTf/VO_*x*_ sample. Red dotted area shows the region of interest. (b–f) Optical microscopy images of the region of interest under DC heating in an X atmosphere, showing the propagation of heat across the notched area prior to failure. The time stamps indicate the time elapsed since the DC heating began.

We carried out a detailed study of Joule heating limits of CNTf/VO_*x*_ as a model system for how and why the CNT failure was occurring in the presence of metal oxides. We used an optical microscope to directly observe failure *in situ* during Joule heating of a notched CNTf/VO_*x*_ sample (Figure 5). The notch ensures that the current density (i.e., current per cross-sectional area) is maximized at the “bridge” between the two sides of the sample so that the optical microscope can observe the failure occurring (Movie S1). We hypothesized that the notch would show a local high current density, which would in turn cause locally high temperatures, leading to mechanical failure, i.e., hotspots cause crack propagation, not vice versa. The glowing spots in the images in Figure 5 are associated with locally high current density and temperature, indicating that local heating occurs first followed by local degradation and mechanical failure propagating across the notch.

**Figure 6:**
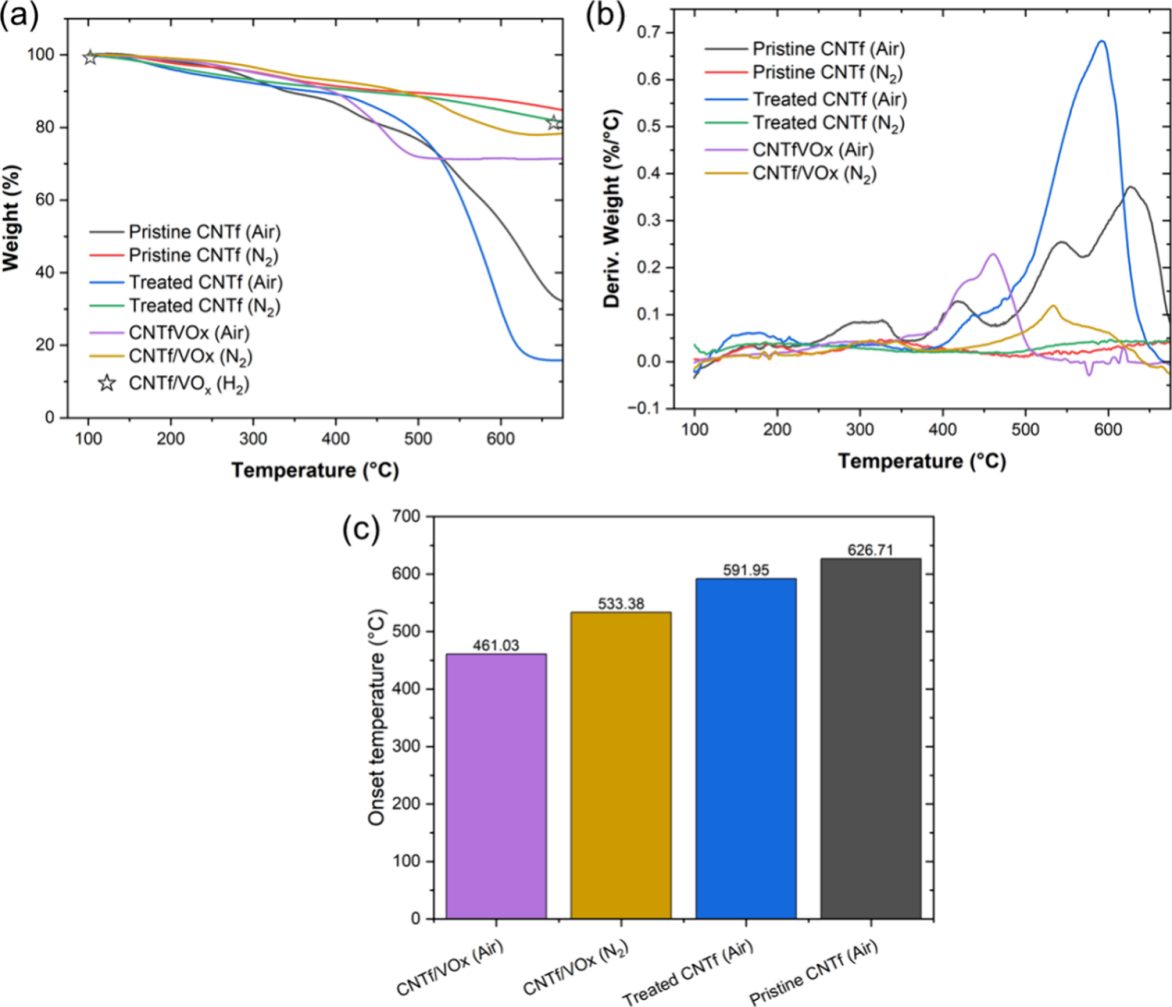
(a) TGA curves for the CNT fabric and the CNTf/VO_*x*_ composite under air and inert (nitrogen) atmospheres. Stars correspond to the mass of the sample heated under hydrogen using a tube furnace. (b) Derivative weight loss with temperature. (c) Shift in oxidation temperatures, from pristine CNTf to treated CNTf and to CNTf/VO_*x*_ (air and inert), is clearly seen.

We then hypothesized the decrease in CNTf mechanical stability at high temperature is caused by a decrease in thermal stability associated with the presence of the metal oxide. To verify this, we carried out TGA measurements on CNTf and CNTf/VO_*x*_ in air and inert atmospheres (Figure 6). The results show that the oxidation onset temperature in air decreases from pristine CNTf to ozone-treated CNTf to CNTf/VO_*x*_. Interestingly, even though the CNTf oxidation is inhibited by an inert atmosphere, the same is not true for CNTf/VO_*x*_ in the same inert atmosphere. This suggests that although the oxidation temperature of CNTf/VO_*x*_ is delayed from ∼461 to ∼533 °C by changing the atmosphere from air to inert, the CNTf still oxidizes in the presence of the metal oxide. In fact, the literature suggests that metal oxides undergo carbothermal reduction in the presence of CNTs, which are oxidized in the process.^20^ The TGA experiments indicate that this carbothermal reduction is indeed the cause of decreased CNTf stability during Joule heating.

To mitigate this decreased stability, we then investigated the effect of a reducing atmosphere on the oxidation of the CNT by the metal oxide. We heated the CNTf/VO_*x*_ sample up to 700 °C in a H_2_ atmosphere in a tube furnace and measured the weight loss (marked as stars in Figure 6). The weight loss was substantially lower than in air or inert, confirming that a reducing atmosphere mitigates the oxidation of the CNTs by the metal oxides. While CNT oxidation likely occurs via the release of lattice oxygen from the surface of the metal oxide, this process is inhibited by the surrounding reducing atmosphere.^20^ Thus, the maximum temperature of metal oxide-CNT is low for air, higher for inert, and higher still for H_2_.

**Figure 7:**
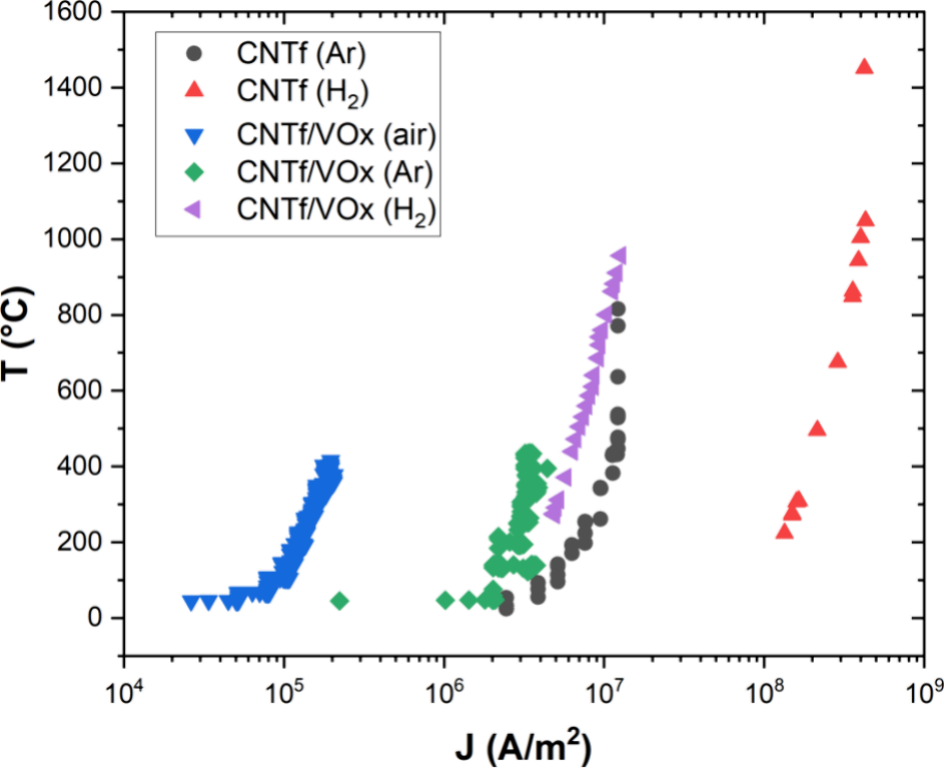
Joule heating experiments showing temperature vs current density. The data show a reducing atmosphere of hydrogen extends the processing window of both CNTf and CNTf/VO_*x*_ to be more stable than in air or inert.

With this new approach for enhancing the stability of CNTf in hand, we then carried out Joule heating in multiple atmospheres and monitored current density, temperature, and resistance (Figure 7, Figure S6). For all the samples, the resistance decreases at low temperatures (semiconducting behavior) and increases at high temperatures (metallic behavior). In Figure 7, the data show that the hydrogen atmosphere resulted in a *T*_failure_ of ∼1000 °C for the CNTf/VO_*x*_ composite, which is even better than that of the pristine CNTf in inert. The *T*_failure_ for CNTf in hydrogen was at least 1500 °C, verifying that the reducing atmosphere helps mitigate the oxidation onset for pristine CNTf as well. (Note that the measurement limits for the pyrometer used in this experiment was 200–1500 °C, and so it could not read below or above this temperature range.) We hypothesize that the inhibition of CNT oxidation also prevents the carbothermal reduction and deterioration of the metal oxides, which we investigate through Raman spectroscopy below.

**Figure 8:**
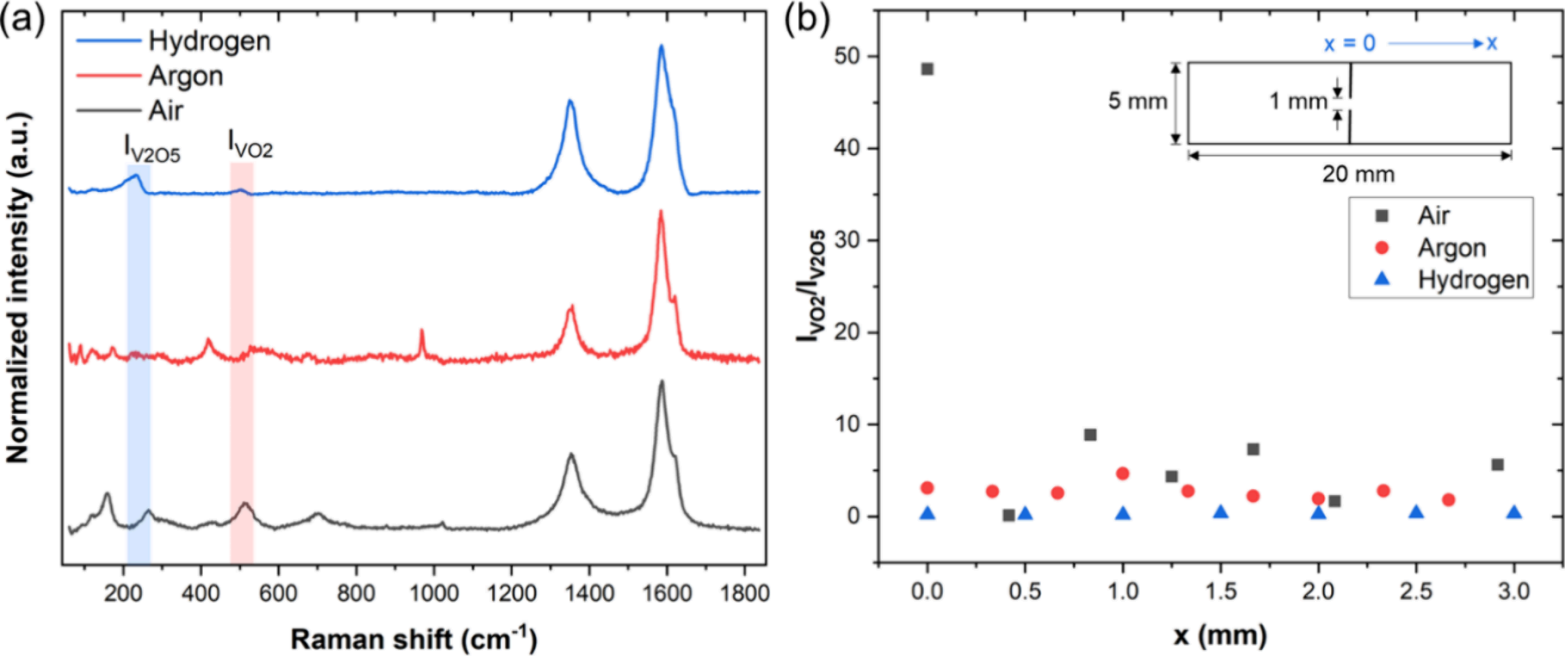
(a) Raman spectra of CNTf/VO_*x*_ samples at the *x* = 2 mm mark, where *x* is measured from the notch. *I*_VO2_ is the peak at ∼500 cm^–1^ corresponding to VO_2_, while *I*_V2O5_ is the peak at ∼200 cm^–1^ corresponding to V_2_O_5_. (b) *I*_VO2_/*I*_V2O5_ ratio as a function of distance from the notch. Inset shows how *x* is defined for the notched CNTf/VO_*x*_ sample. Note that in air, a high degree of reduction is seen near the failure point, indicating that the inorganic phase is catalyzing CNT oxidation.

Finally, to verify the mitigation of CNT oxidation in hydrogen, we compared the Raman spectra of the samples Joule-heated to failure in the different atmospheres mentioned above (Figure 8). For all the samples, we compared the peak intensity ratio of a relatively reduced phase (VO_2_) to that of a relatively oxidized phase (V_2_O_5_) and found a higher degree of metal oxide reduction in air compared to argon or hydrogen. In fact, the highest level of reduction was seen near the failure point (at the notch) for the sample heated in air. This is likely because the metal oxide got reduced while catalyzing the CNT oxidation in the process. This finding is important because it shows that the most oxidizing atmosphere results in the most CNT oxidation while sacrificing the lattice oxygen from the metal oxide. Notably, the metal oxide Raman peaks are still evident in the sample heated in hydrogen, suggesting that a controlled atmosphere free of oxygen or water can prevent the degradation of not only the CNTs but also the metal oxides in the presence of carbon.

## 4 Conclusions

To summarize, we utilized Joule heating to induce controlled crystallization of two metal oxide matrices grown on CNT networks, with a view toward applications as battery electrodes, catalysts, and sensors. We found that the metal oxide matrix affects the thermal stability and Joule breakdown of the CNTs, even in inert atmospheres; at high temperatures (either from oven or Joule heating), the metal oxide undergoes carbothermal reduction, simultaneously oxidizing the CNTs. This finding is consistent with a prior study on the catalytic effects of metal oxides on the oxidation resistance of CNT/inorganic hybrids. We then demonstrated that a reducing atmosphere such as hydrogen can help mitigate the CNT oxidation and thus extend the thermal stability and Joule heating processing window of the CNTf/inorganic composite to ∼1000 °C. While this work focused on composites of CNTf with two metal oxides (namely, MnO_*x*_ and VO_*x*_), we envision that our approach, combining reduced annealing time with high processing temperatures, may help thermally process industrially important nanocarbon/inorganic composites (for example, CNT composites with ZnO, TiO_2_, NiO, Co_3_O_4_, Al_2_O_3_, and so on) that are otherwise difficult or time- and energy-consuming.

## Supporting Information Available

Digital video of the failure propagation mechanism, picture of Joule heating setup in hydrogen atmosphere, resistance–temperature profiles for CNTf/MnO_*x*_ during Joule heating and cooling, WAXS patterns for CNTf/VO_*x*_, comparison of temperature–time profiles for CNTf/MnO_*x*_ and CNTf Joule-heated to failure, SEM images of CNTf/MnO_*x*_ and CNTf Joule-heated to failure, current density vs normalized resistance for Joule-heated samples in different atmospheres, current density vs normalized resistance for different CNT fabrics (PDF)
