# Supplementary material for: Joule Heating in Controlled Atmospheres to Process Nanocarbon/Transition Metal Oxide Composites and Electrodes
Source: ACS Appl Nano Mater. 2024 Jun 14;7(12):14557–65. doi: 10.1021/acsanm.4c02081 (PMC11217916; doi:10.1021/acsanm.4c02081)
Supplement: Supplementary file 1 — an4c02081_si_001.pdf [file an4c02081_si_001.pdf]

Supporting Information

Joule Heating in Controlled Atmospheres to Process Nanocarbon/Transition Metal Oxide Composites and Electrodes

Shegufta Upama, [1,2] Luis Arevalo,[2] Afshin Pendashteh,[2] Anastasiia Mikhalchan,[2] Micah J. Green*,[1,3] and Juan Jose Vilatela.*[2]

[1]Department of Materials Science & Engineering, Texas A&M University, College Station, TX 77843, USA.

[2]IMDEA Materials Institute, 28906 Getafe, Madrid, Spain.

[3]Artie McFerrin Department of Chemical Engineering, Texas A&M University, College Station, TX 77843, USA.

*Corresponding authors

Email: juanjose.vilatela@imdea.org, micah.green@tamu.edu

A video of the failure propagation mechanism under optical microscopy is included as a separate file (**Movie S1**).

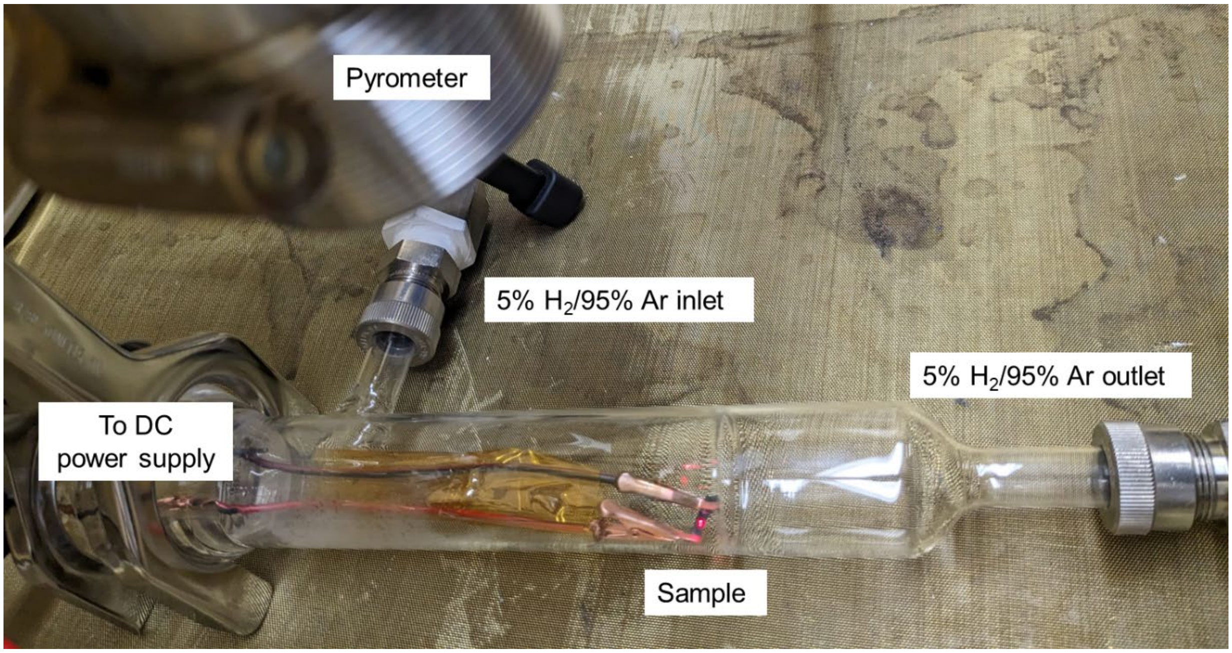

Figure S1: Joule heating setup in hydrogen atmosphere. Note that Kapton tape was used to fix the electrodes in place. The electrodes, which hold the sample, are connected to a DC power supply.

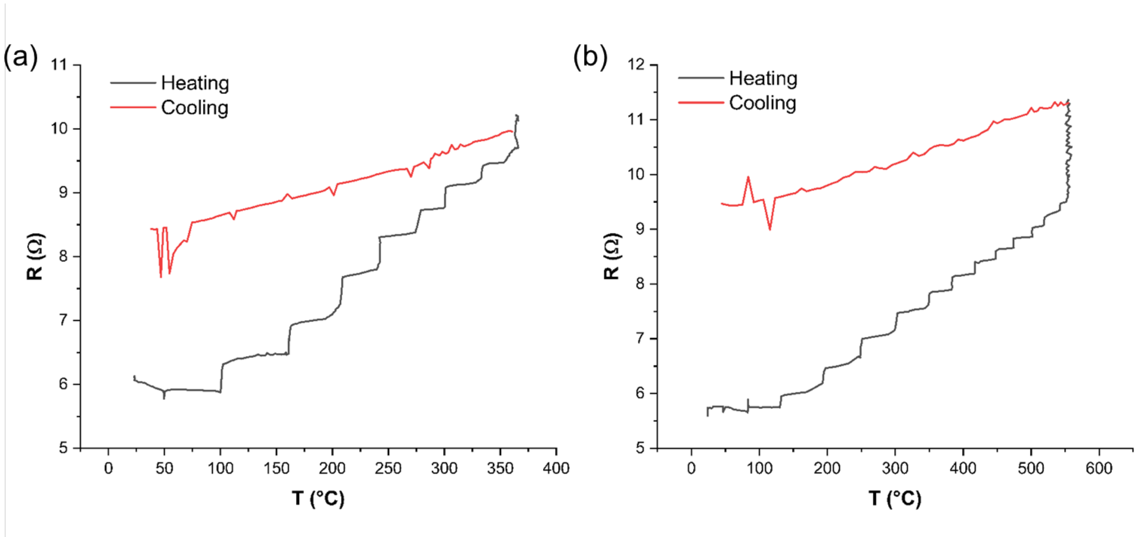

Figure S2: Irreversible increase in resistance on heating CNTf/MnO_x to (a) 350 °C, and (b) 550 °C, due to phase change and shrinkage of the metal oxide. Note that when the temperature is held constant for 10 min, the resistance continues increasing.

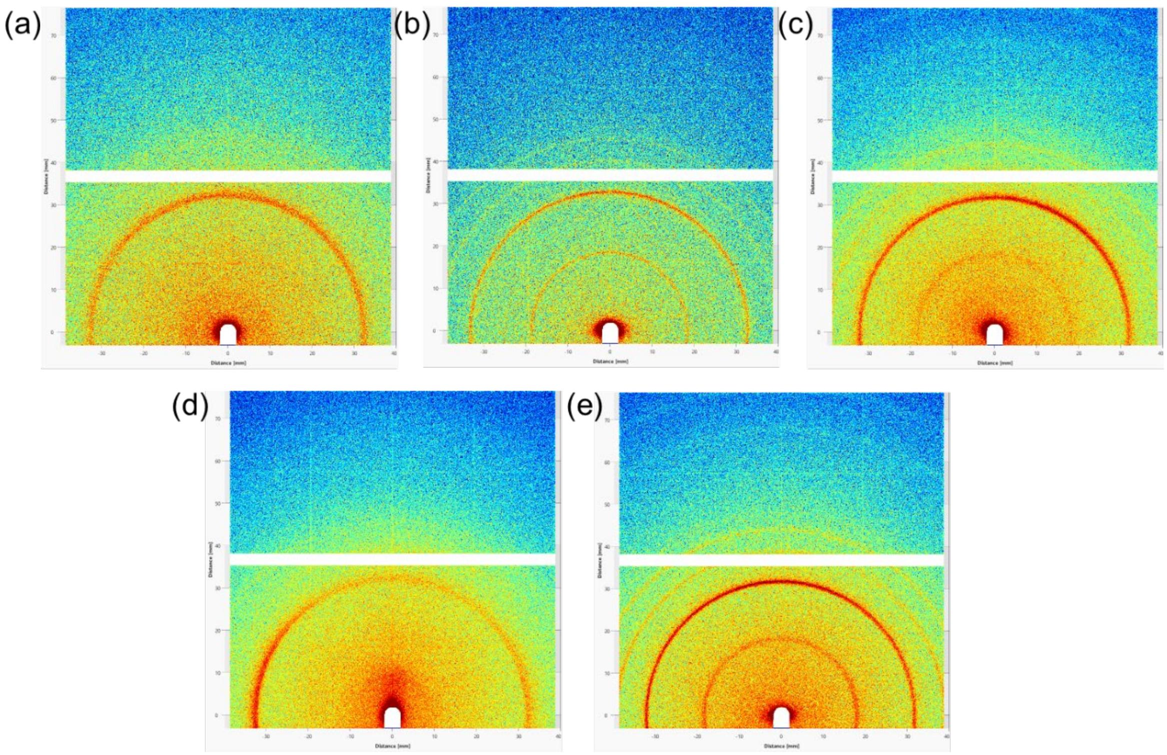

Figure S3: Raw data for the WAXS patterns: (a) As-deposited CNTf/VO_x, (b) CNTf/VO_x oven-heated to 350 °C in air, (c) CNTf/VO_x DC-heated to 350 °C in air, (d) CNTf/VO_x DC-heated to 350 °C in argon, and (e) CNTf/VO_x DC-heated to 350 °C in argon and then air (halfway through).

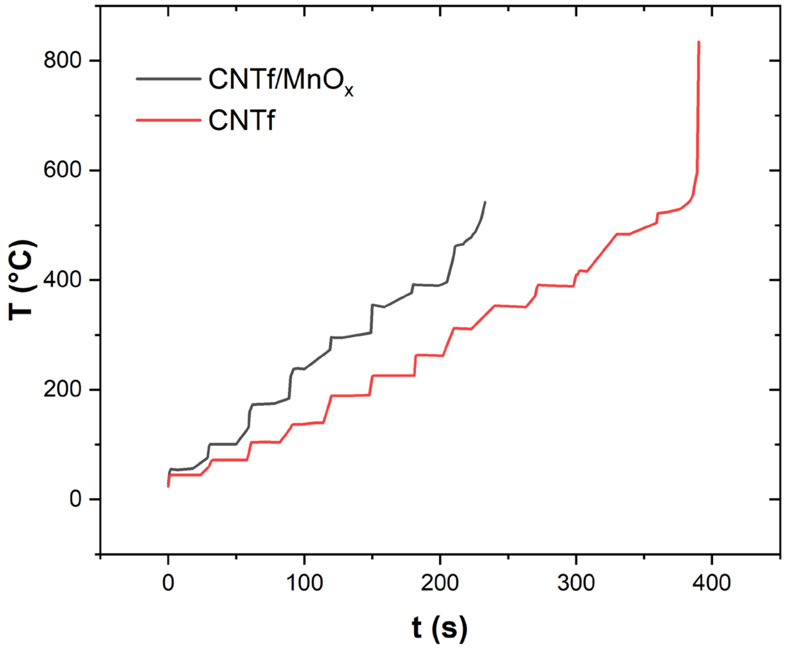

Figure S4: Temperature-time data for CNTf and CNTf/MnO_x samples that were Joule-heated to failure in argon

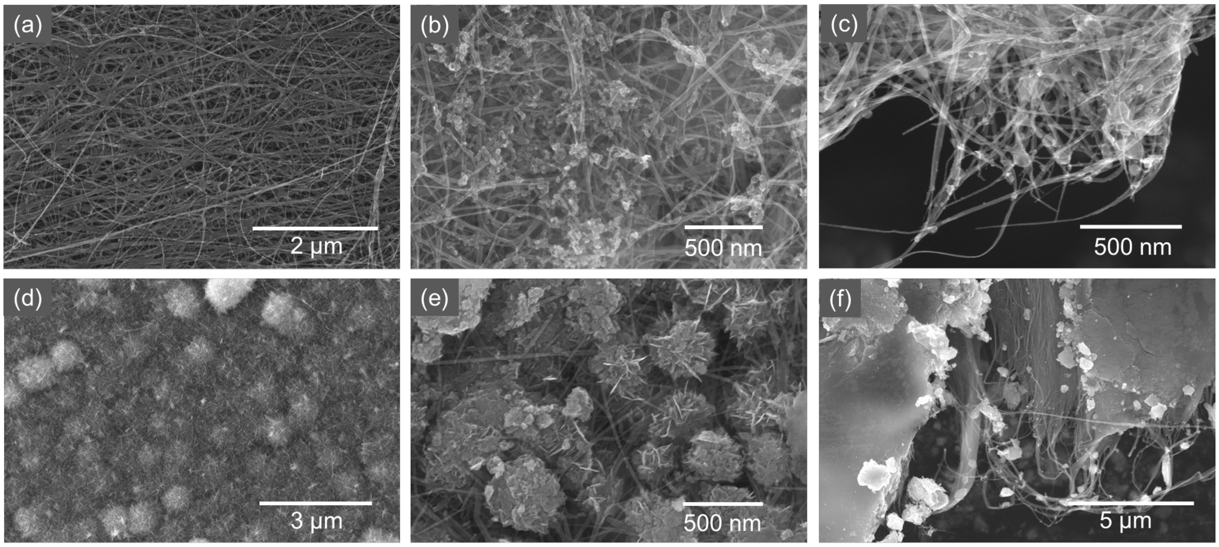

Figure S5: SEM images of CNTf and CNTf/MnO_x samples that were Joule-heated to failure in argon: (a) CNTf away from the fracture surface, and (b, c) CNTf close to the fracture surface, showing CNT fiber breakage and clusters of carbonaceous structures formed due to the locally high temperature; (d) CNTf/MnO_x away from the fracture surface, and (e, f) CNTf/MnO_x close to the fracture surface, showing CNT fiber pullout and agglomeration of MnO_x nanoflowers.

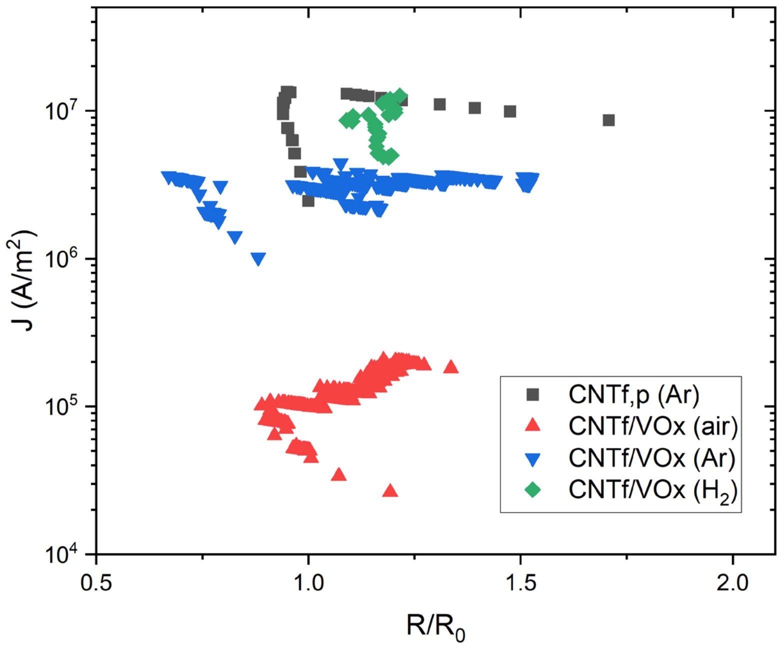

Figure S6: Current density vs. normalized resistance for Joule-heated CNTf/VOx in different atmospheres and Joule-heated CNTf in inert.

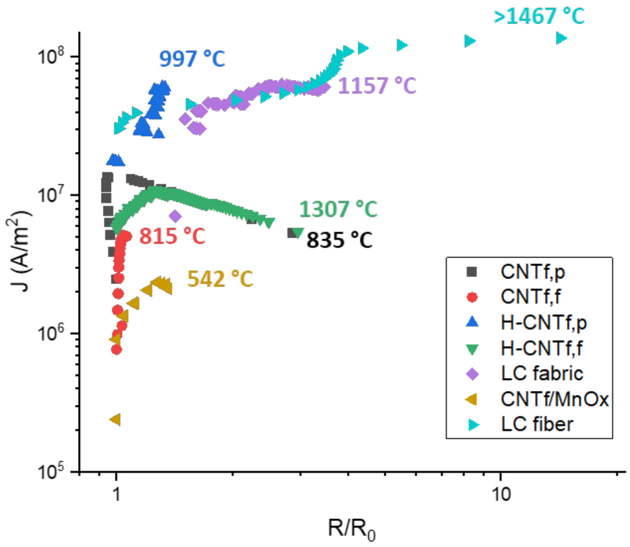

Figure S7: Current density vs. normalized resistance for different CNT fabrics: in-house CNT fabrics (CNTf, pristine and functionalized), commercial CNTf from Huntsman that was directly grown from the gas phase by FC-CVD (H-CNTf, pristine and functionalized), commercial CNTf from DexMat that was spun from liquid crystalline solution (LC fabric). Note that the data for LC fiber was taken from REF[1].

References

[1] Wang, X.; Behabtu, N.; Young, C. C.; Tsentalovich, D. E.; Pasquali, M.; Kono, J. “High-Ampacity Power Cables of Tightly-Packed and Aligned Carbon Nanotubes,” *Adv. Funct. Mater.*, vol. 24, no. 21, pp. 3241–3249, 2014, doi: 10.1002/adfm.201303865.
